# Earthworm coelomocytes as nanoscavenger of ZnO NPs

**DOI:** 10.1186/1556-276X-9-259

**Published:** 2014-05-23

**Authors:** Shruti Gupta, Tanuja Kushwah, Shweta Yadav

**Affiliations:** 1 Department of Zoology, School of Biological Sciences, Dr H S Gour Central University, Sagar, MP 470003, India

**Keywords:** Coelomocytes, *Eisenia fetida*, Nanoscavenger, Biotransformation, Chloragocytes, Internalization

## Abstract

Earthworms can ‘biotransform’ or ‘biodegrade’ chemical contaminants, rendering them harmless in their bodies, and can bioaccumulate them in their tissues. They ‘absorb’ the dissolved chemicals through their moist ‘body wall’ due to the interstitial water and also ingest by ‘mouth’ while soil passes through the gut. Since the advent of the nanotechnology era, the environmental sink has been continuously receiving engineered nanomaterials as well as their derivatives. Our current understanding of the potential impact of nanomaterials and their natural scavenger is limited. In the present investigation, we studied the cellular uptake of ZnO nanoparticles (NPs) by coelomocytes especially by chloragocytes of *Eisenia fetida* and their role as nanoscavenger. Results from exposure to 100- and 50-nm ZnO NPs indicate that coelomocytes of the earthworm *E. fetida* show no significant DNA damage at a dose lower than 3 mg/l and have the potential ability to uptake ZnO NPs from the soil ecosystem and transform them into microparticles.

## Background

The coelomic fluid, haemolymph and blood in some phyla (Nemertea, Annelida) of invertebrates play a crucial role in physiological processes, *viz*., transportation of nutrients, metabolic intermediates and end products, respiratory gases and signalling molecules. These body fluids have a defined composition, containing characteristic cell types which take part in blood coagulation, wound healing and immune response. The cells of invertebrate body fluids are analogous in function with vertebrate blood cells. Therefore, we need to understand the influence of nanoparticles (NPs) and their cytotoxicity and genotoxicity.

In this context, some earlier studies suggested the contribution of coelomocytes to homeostatic regulation, e.g. in blood coagulation immune reactions and in regeneration of lost body parts. Annelids are the first animals in the phylogenetic tree in which not only the cellular but also the humoral immune response is developed. During the cellular immune response, coelomocytes play a role in phagocytosis, inflammatory processes, graft rejection and coagulation of coelomic fluid. During the humoral immune response, they secrete lysozyme, agglutinin, peroxidase, phenoloxidases and antimicrobial factors (fetidin, lysenin, eiseniapore, coelomic cytolytic factor). Cytotoxic molecules may increase the intracellular calcium concentration in target cells, which participate in exocytosis, enzyme function, regulation of gene expression, cell proliferation and apoptosis; therefore, chloragocytes can induce and influence important physiological processes by these signal molecules [1]. Thus, they play a remarkable role in the function of the earthworm immune system and are involved in phagocytosis and the release of lytic factors which are characteristics of innate immunity [2].

Earthworms have pores that connect the coelomic cavity to the exterior, through which cells are extruded following stress. These cells are considered as immune cells (type of leucocytes) that have long been considered to constitute the major innate immune defense system of annelids [3,4]. Coelomocytes from various sources have shown to be capable of phagocytosis and thus perform functions of macrophages. These have natural killer cell features, mediate lytic reactions against several targets and also secrete antimicrobial peptides [5-9]. Valembois et al. [10] classified coelomocytes into three major categories: acidophils, basophils and chloragocytes (chloragogen cells or eleocytes). These cells contain characteristic granules called chloragosomes which are thought to be involved in the protection of cells and organisms against foreign substances [11,12].

Immunity is a vital function to maintain an organism’s well-being and represents a sensitive physiological indicator that may be affected even at low concentrations of nanomaterial exposure. Only a handful of studies exist so far to aid the current understanding of immune responses to nanomaterials in invertebrates, particularly earthworms. This includes the *in vitro* study on *Eisenia fetida* exposed to silver nanoparticles (AgNPs) [2] supporting molecular responses observed *in vivo*[13] and studies on other earthworm species by Vander Ploeg and coworkers where *Lumbricus rubellus* was exposed to the carbon-based nanoparticle C_60_ fullerene *in vivo* (2011) and *in vitro* (2012). Carbon-based nanomaterials can affect the life history traits of *Eisenia veneta*[14], *E. fetida*[15] and *L. rubellus*[16]. Peterson et al. [17] also reported bioaccumulation of C_60_ fullerenes in *E. fetida* and in *Lumbriculus variegatus*. Cholewa et al. [18] proved the internalizing property of coelomocytes of *L. rubellus for* polymeric NPs (hydrodynamic diameter of 45 ± 5 mm) apparently involving energy-dependent transport mechanisms (clathrin- and caveolin-mediated endocytosis pathways) [19]. These studies are only indicative of the extent to which nanomaterials may interfere with the function of the earthworm’s immune system.

Manufactured NPs have a wide range of applications, having unique properties as compared with their bulk counterparts [20]. Estimation of the worldwide investment in nanotechnology previews that US$3 trillion will be attained in 2014 [21]. However, there is a growing concern regarding the safety of NPs for their toxicity. Several studies have reported the potential risk to human health from NPs based on evidences of inflammatory reaction by metal-based NPs [22]. Recent studies however suggest that NPs may be released from these products through normal use and then enter in waste water streams [23]. A significant portion of NPs in waste water is expected to partition to sewage sludge [24,25]. Depending on local practices, varying proportions of sewage sludge are disposed of in landfills, incinerated or applied to agricultural lands as biosolids. Therefore, terrestrial ecosystems are expected to be an ultimate sink for a larger portion of NPs [26].

This raises concern about the potential of NPs for ecological effects, entry into the food web and ultimately human exposure by consumption of contaminated agricultural products. Therefore, it is of great interest to determine if intact NPs can be taken up by organisms from soil. Since not much work has been carried out in this direction regarding the uptake of these NPs and to find out the natural scavengers, the present investigation was done to study the influence and cellular uptake of NPs by coelomocytes of the model detritivore *E. fetida* (Savigny, 1826) by using ZnO NPs (next-generation NPs of biological applications including antimicrobial agents, drug delivery, bioimaging probes and cancer treatment). Our objective was to understand the influence of these NPs on coelomocytes of *E. fetida* and the underlying mechanisms in order to provide more information on their uptake in the soil ecosystem.

## Methods

### Experimental animal

Adult earthworms *E. fetida* (Savigny, 1826) were collected from Vermiculture Research Station, DS College (Dr BRA University), Aligarh, India, and were assimilated in an experimental chamber without light, at low temperature (approximately 24°C), and kept in earthworm beddings. The worms were acclimated for 2 weeks before cell collection following Brousseau et al*.*[27] with regular feeding.

### Extrusion of coelomocytes

Earthworm coelomocytes were collected using a non-invasive method following [28-30]. Briefly, each worm was rinsed in cold water and placed on a paper towel. One fourth of the posterior part was massaged to expel the content of the lower gut. Then, each worm was placed for 3 min in a 15-ml polypropylene tube containing 30 ml of cold extrusion medium [Nacl (71.2 mM), EDTA disodium salt (6.7 mM), GGE (50.4 mM), ethanol (2% *v*/*v*) and a supplement of antibiotic and antimycotic agents: penicillin G sodium salt (100 U/ml), streptomycin sulphate (100 μg/ml), amphotericin B (25 mg/ml)]. Ethanol (5%) was added to the extrusion medium immediately before cell extrusion. After 3 min, the worm was removed and the volume was made up to 12 ml by adding ice-cold Ca-free Luria Broth Agar Media containing 1.5 mM NaCl, 4.8 mM KCl, 1.1 mM MgSO_4_ · 7H_2_O, 0.45 M KH_2_PO_4_, 0.3 mM Na_2_PO_4_ · H_2_O and 4.2 mM NaHCO_3_ adjusted to pH 7.3 and osmolarity adjusted to 300 mosM [27]. Finally, the cells were re-suspended in Ca-LBSS (containing 3.8 mM CaCl_2_) and loaded in a culture plate with Dulbecco’s Modified Eagle Medium (DMEM) supplement with foetal bovine serum. The selected choloragocytes were subjected to subculturing.

### Viability determination

The cell viability was determined by both trypan blue staining and flow cytometry. In this case, 5 μl of a 1 mg/l propidium iodide solution was added to 500 μl of cell suspension and the fluorescence measured in FL3.

### Exposure of ZnO NPs

Chloragocytes were seeded into a 96-well plate at 5 × 10^5^ cells/ml and treated with ZnO NPs (for 3, 6, 12, 24 and 48 h) of diameters 100 and 50 nm (0.5, 1.0, 2.0, 3.0, 4.0 and 5.0 mg/l). ZnO NPs were purchased from Sigma-Aldrich (St. Louis, MO, USA), and their morphology and size were examined by transmission electron microscopy (TEM) at The Energy Research Institute, New Delhi, India.

### DNA damage analysis

**Figure 1:**
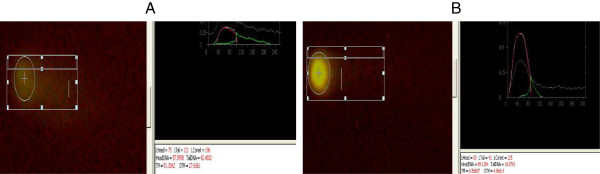
DNA damage of coelomocytes (A) in the control and (B) after exposure to 100-nm NPs (3 mg/l).

The Comet assay was performed as described by Singh et al*.*[31]. Ethidium bromide-stained nuclei were examined with a fluorescent microscope (Leica Microsystems, Wetzlar, Germany). Images were analyzed with the software CASP according to the method of Collins et al*.*[32] (Figure 1).

### Statistical analysis

Results are the means of three replicates. Two-way analysis of variance (ANOVA) was performed by using the SPSS 10.5 software.

## Results and discussion

**Figure 2:**
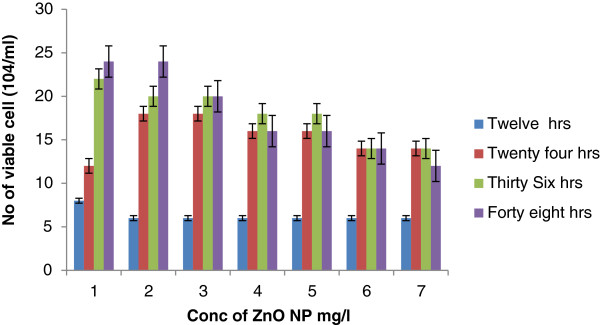
Viability of coelomocytes after exposure to ZnO NPs (100 nm) at different intervals.

The total viability of coelomocytes after exposure to 100-nm ZnO ranged from 6 ± 1.02 to 24 ± 3.12 × 10^4^/ml (Figure 2). At 12 h of exposure, the highest viability of cells was recorded: 6 ± 10.03 × 10^4^/ml, which was consistently the same in all concentrations of exposure. However, at 24 h of exposure, the highest viability (18 ± 2.14 × 10^4^/ml) was recorded at the doses of 0.5 and 1.0 mg/l and the total cell count decreased from 16 ± 2.01 × 10^4^/ml to 14 ± 1.02 × 10^4^/ml at exposure of 2 to 5 mg/l ZnO NPs. This reflects that at high concentration the viability of coelomocytes decreases significantly. Similarly, at 36 h of exposure of up to 1 mg/l, the viability of coelomocytes recorded was 20 ± 2.01 × 10^4^/ml, and this was gradually decreased (14 ± 2.01 × 10^4^/ml) by increasing the concentration of nanoparticles. At 48 h, the number of coelomocytes was similar to that of control (24 ± 2.12 × 10^4^/ml) at 0.5 mg/l but gradually decreased with the increase in the concentration of nanoparticles. Results indicate that the viability of coelomocytes deceases with the increase in the concentration of NPs (100 nm).

**Figure 3:**
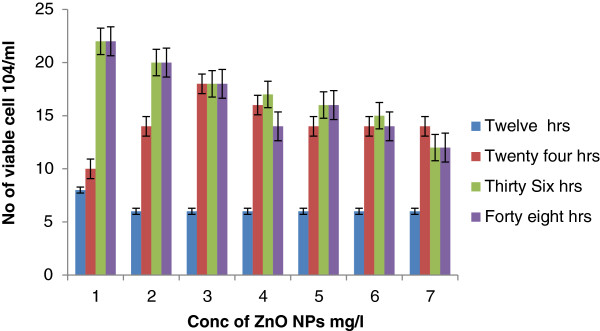
Viability of coelomocytes after exposure to ZnO NPs (50 nm) at different intervals.

**Figure 4:**
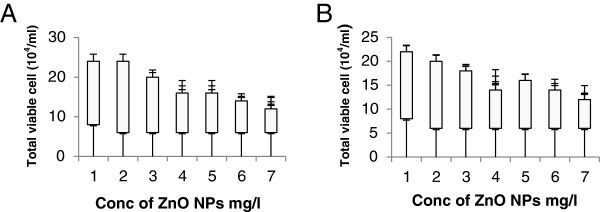
Total viability of coelomocytes after exposure to ZnO NPs: (A) 100 nm and (B) 50 nm.

After exposure to 50-nm ZnO at 12 h, the viability recorded was 6 ± 1.0× 10^4^/ml which was dependent on neither the size nor the concentration of NPs. However, at 24 h, the uptake of NPs triggers cell replication and increases the number of coelomocytes from 10 ± 2.04 × 10^4^/ml to 18 ± 3.12 × 10^4^/ml (Figure 3). However, there was a little trend in the decrease in the number of coelomocytes: 14 ± 1.12 × 10^4^/ml. At 48 h, the highest cell count was recorded at exposure of 0.5 mg/l. There was a gradual decrease in coelomocytes (18 ± 2.08 × 10^4^/ml to 12 ± 1.06 × 10^4^/ml). However, the total viability ranges were between 6 ± 1.02 × 10^4^/ml and 20 ± 3.12 × 10^4^/ml. Results indicate that exposure up to 1 mg/l increases the replication of coelomocytes (Figure 4). Yang et al. [33] also recorded the uptake of NPs which depends on their size and concentration.

Earthworms in general are tolerant to many chemical contaminants including heavy metals and organic pollutants in soil and can bioaccumulate them in their tissue [34]. They absorb the dissolved chemicals through their moist body wall due to the interstitial water and also ‘ingest’ by mouth while the soil passes through the gut. They either ‘biotransform’ or ‘biodegrade’ chemical contaminants, rendering them harmless in their bodies. Satchell [35] suggested that earthworms can uptake chemicals from soil pore water through passive ‘absorption’ of the dissolved fraction through their body wall. Coelomic uptake can also occur as soil is ingested and passed through the coelomic cavity. Earthworms apparently possess a number of mechanisms for uptake, immobilization and excretion of heavy metals and other chemicals. However, the internalization mechanisms and intracellular trafficking of NPs require further study. This study examined the intracellular localization and subsequently the uptake mechanism. After 6 h, the uptake of 50-nm NPs was higher than that of 100-nm NPs. Smaller sized NPs were distributed throughout the whole cell. However, 100-nm NPs were mostly co-localized with endosomes, indicating that the cellular uptake was associated with endosomes. After 12 h of exposure, the cellular uptake of 50-nm NPs was still higher than that of 100-nm NPs while localization of 100-nm NPs decreased and the fluorescence of NPs was dispersed throughout the chloragocyte, suggesting that NPs might escape from endosomes into the cytoplasm or be resorted to other organelles [36]. However, some metals are taken up by earthworms and bound by proteins called ‘metallothioneins’ which have the capacity to bind metals. Ireland and Richards [37] found that cadmium and lead are concentrated in the chloragogen cells of earthworms.

**Figure 5:**
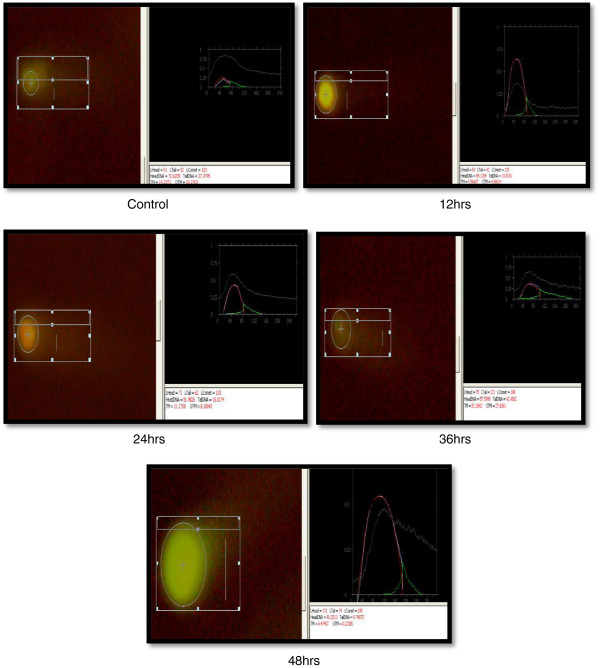
Comet assay of coelomocytes after exposure to 50-nm ZnO NPs (3 mg/l) at different intervals.

**Figure 6:**
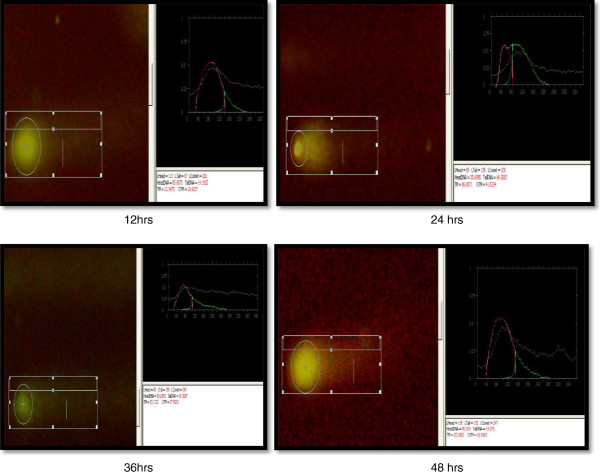
Comet assay of coelomocytes after exposure to 50-nm ZnO NPs (5 mg/l) at different intervals.

**Figure 7:**
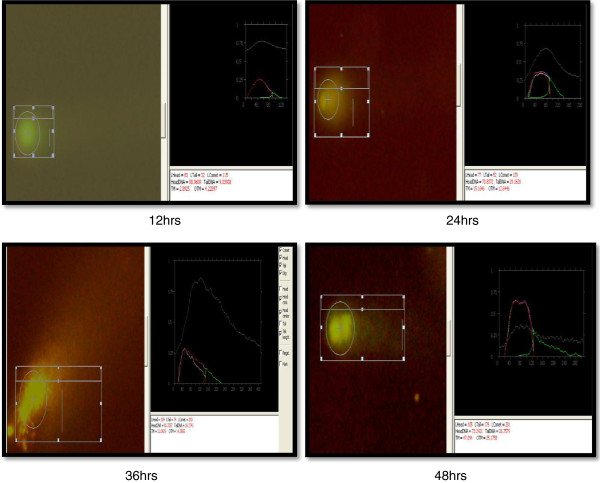
Comet assay of coelomocytes after exposure to 100-nm ZnO NPs (5 mg/l) at different intervals.

**Figure 8:**
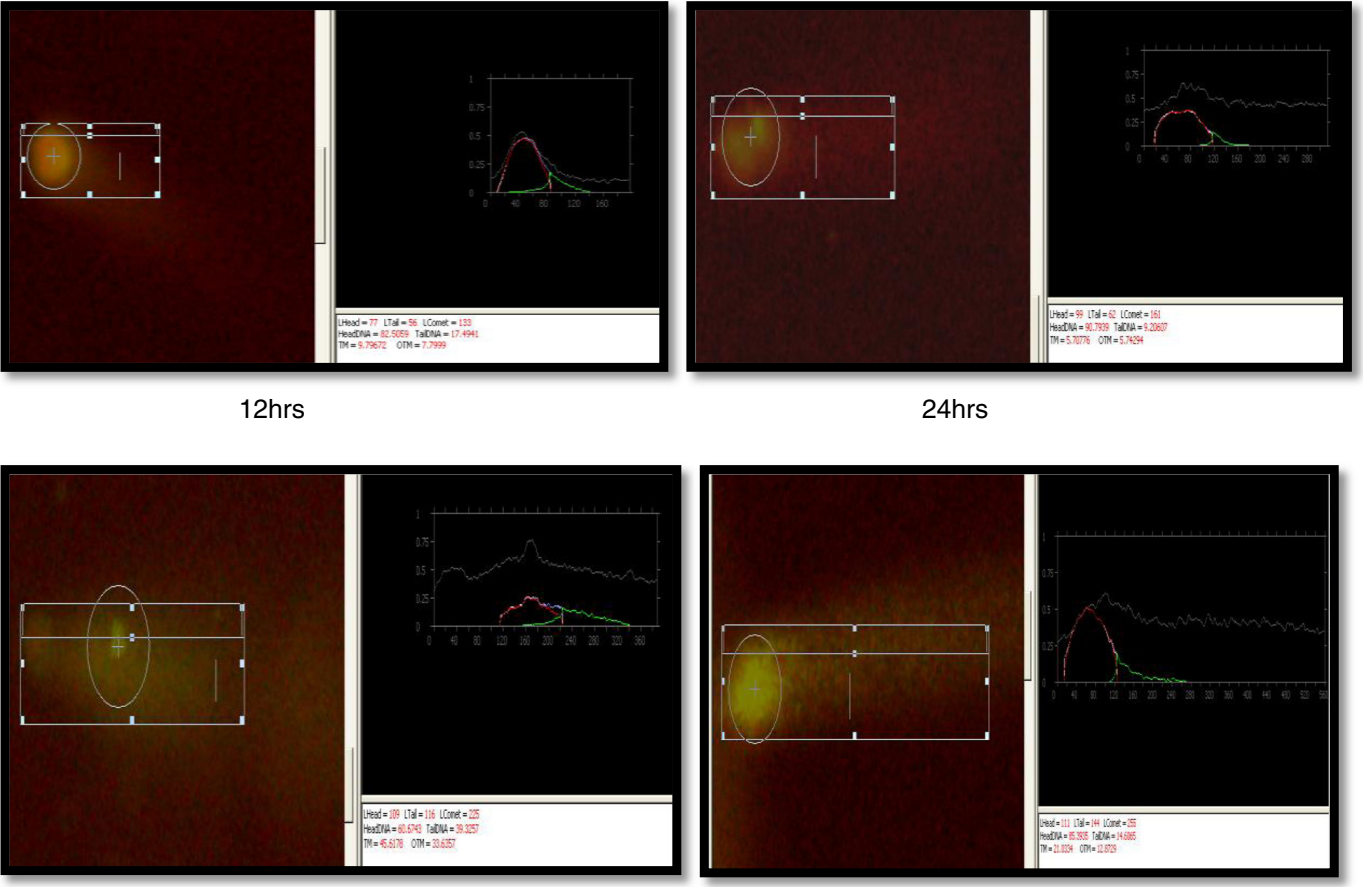
Comet assay of coelomocytes after exposure to 100-nm ZnO NPs (3 mg/l) at different intervals.

Comet, tail DNA and Olive tail moment (OTM) were chosen to evaluate DNA damage in coelomocytes of *E. fetida* after exposure to 100- and 50-nm ZnO NPs at 1.0, 3.0 and 5.0 mg/l at different intervals (12, 24, 36 and 48 h). Results are shown in Table 1 and Figures 5, 6, 7 and 8. Coelomocytes exhibited DNA damage when exposed to 100-nm ZnO NPs at 36 and 48 h at the doses of 3.0 and 50 mg/l, while up to 24 h, there was no significant DNA damage. After exposure to 50-nm ZnO NPs at the dose of 3.0 mg/l, coelomocytes showed significant DNA damage at 40 h, and at 5.0 mg/l, significant Olive tail moment of comet was recorded at 36 and 48 h. However, no DNA damage was observed when the exposure dose was 1.0 mg/l for both 100- and 50-nm ZnO NPs. The results of the comet assay have shown clearly that the earthworm coelomocytes experienced DNA damage at exposure of more than 3 mg/l after 24 h. The study corroborates the finding of Bystrzejewska-Piotrowska et al. [38] who observed the capability of earthworms to extract zinc from soil exposed to ZnO nanoparticles. Cholewa et al. [18] demonstrated the capability of internalizing polymeric NPs (hydrodynamic diameter 45 ± 5 nm) by free circulating amoebocytes of the earthworm *L. rubellus* apparently involving an energy-dependent transport mechanism (clathrin and caveolin-mediated endocytosis pathways). Although NP uptake mechanisms are largely unknown in coelomocytes, uptake probably occurs by macropinocytosis [39]. In mammals, macropinocytosis initiates with cell membrane ruffling via actin rearrangement, suggesting an intriguing possibility of passive uptake of NPs that are membrane-adhered. Amongst invertebrates, ascidian haemocytes are able to engulf particles via RGD motif-dependent macropinocytosis [40]. However, such mechanisms are not yet known in earthworms. Another potential phagocytic pathway is via scavenger receptors that are expressed by both human macrophages and macrophage-like THP-1 cells [39]. Scavenger receptors are a conserved pattern known to bind lipids and polyanions for phagocytosis. In invertebrates, haemocytes from insects [41] and molluscs [42] are known to affect the scavenger receptor-mediated uptake of pathogens and apoptotic cells. To date, scavenger receptors are yet to be identified in earthworms; however, their ubiquitous presence suggests an unequivocally conserved role in innate immune recognition that may be involved in NP uptake as in the vertebrate counterpart. The coelomic fluid of earthworms is sometimes assumed, in the immunological context, to be equivalent to blood plasma in mammals, both representing a protein-rich immune-competent circulatory system. Distinct from the mammalian counterpart is the existence of chloragocytes involved in the regulation of essential minerals, haemoglobins and metallothioneins in response to natural stressors [43]. This is probably by functional analogy with the hepatic/renal systems of vertebrates, and chloragocytes may contribute to regulation of the total protein balance in coelomic fluid.

**Table 1:** **DNA damage after exposure to ZnO NPs on coelomocytes of *Eisenia fetida *** **at different intervals**

**Serial number**	**Dose (mg/ml)**	**Size of NPs (nm)**	**Time (h)**	**Head**	**Tail**	**Comet**	**Head DNA**	**Tail DNA**	**Tail moment**	**Olive tail moment**
1	0.0	Nil	0	51	52	103	72.62	27.37	14.23	10.27
2	1.0	100	12	50	51	104	72.62	26.43	14.12	10.17
3	1.0	100	24	51	52	103	72.61	27.32	14.13	10.17
4	1.0	100	36	52	53	104	72.51	27.03	14.23	10.23
5	1.0	100	48	51	52	104	72.61	27.31	14.34	11.23
6	1.0	50	12	50	51	104	72.62	26.43	14.12	10.17
7	1.0	50	24	51	52	102	71.12	27.32	14.13	10.17
8	1.0	50	36	52	53	104	72.51	27.03	14.23	10.23
9	1.0	50	48	51	52	104	72.61	27.31	14.34	11.23
10	3.0	100	12	77	56	133	82.5	17.49	9.79	7.79
11	3.0	100	24	111	144	255	85.39	18.62	21.03	12.82
12	3.0	100	36	105	176	281	73.24	26.75	57.04	25.17
13	3.0	100	48	109	116	225	60.67	39.32	45.6	33.83
14	3.0	50	12	83	42	125	89.12	10.87	4.56	4.66
15	3.0	50	24	71	62	133	81.98	18.01	11.17	8.18
16	3.0	50	36	71	74	245	91.25	18.74	6.47	8.23
17	3.0	50	48	75	121	296	57.59	42.41	51.3	27.63
18	5.0	100	12	83	32	115	90.96	9.03	2.89	4.22
19	5.0	100	24	77	52	129	70.83	15.16	15.16	12.64
20	5.0	100	36	129	74	203	83.72	16.27	12.04	14.34
21	5.0	100	48	105	176	281	73.24	26.75	47.09	25.17
22	5.0	50	12	113	87	200	85.8	14.19	12.34	10.42
23	5.0	50	24	115	132	247	80.92	19.07	25.18	16.43
24	5.0	50	36	85	155	240	65.69	34.32	53.17	27.82
25	5.0	50	48	65	135	242	35.69	64.31	86.8	41.53

In general, toxicological implications arising from selective cellular uptake of nanomaterials are profound. Metal-based nanomaterials readily dissolve and liberate bioactive metal ions and react with biomolecules (proteins and DNA) of the cellular components in a similar manner as a reactive oxygen species (ROS). NPs and free ions co-exist extracellularly and/or intracellularly, indicating a multitude of stress pathways [33,44]. The intracellular uptake of ZnO NPs is likely to involve subsequent fusion with lysosomes that may accelerate the oxidative dissolution of ZnO NPs as indicated in the present study. This implies that ZnO NPs may have targeted impact on coelomocytes as a result of preferential accumulation and subsequent *in situ* molecular damages by liberated Zn^+^ ions [2] at higher concentration. Time course profiling of representative gene expressions, in parallel with flow cytometric analysis of the intracellular ROS level, favours the view that coelomocyte populations are under oxidative stress that can signal-transduce to immune cascades downstream [13]. Recently, coelomocytes were found to recruit calcium for activation [45], and they may possess similar biochemistry to that of calcium and similar signalling to that in higher organisms, linking stress responses to activation of immune systems [46].

## Conclusions

In light of our current understanding of nanomaterial uptake, the present investigation was carried out. The phagocyte population of coelomocytes seems to be a susceptible target of nanomaterials. To evaluate the cellular uptake of ZnO NPs by coelomocytes of earthworm in the soil ecosystem, cell viability with comet assay for genotoxicity investigation was observed. The results from these aspects showed the following: (i) Coelomocytes were viable after exposure to 100- and 50-nm ZnO NPs (up to exposure of 5 mg/l). However, there was a decrease in viability when the exposure dose was 3 mg/l particularly at 48 h. (ii) Exposure to 50-nm NPs triggered the replication of coelomocytes which may be due to the high rate of internalization of NPs. (iii) Exposure to 100- and 50-nm ZnO NPs did not show any significant DNA damage up to exposure less than 3 mg/l. (iv) Coelomocytes effectively uptake the 100- and 50-nm ZnO NPs up to 3 mg/l exposure dose within 24 to 36 h without causing any significant DNA damage. The study explicitly implies the NP recognition involved in cellular uptake as well as sub- and inter-cellular events that may uncover further intriguing insights into the earthworm as nanoscavenger.

## Competing interests

The authors declare that they have no competing interests.

## Authors’ contributions

SG designed the experiment, analysed the data and was involved in drafting the manuscript. TK replicated the experiment and statistically analysed the data. SY gave the final approval for publication. All authors read and approved the final manuscript.

## References

[B1] Hanley C Thurber A Hanna C Punnoose A Zhang J Wingett DG The influence of cell type and ZnO nanoparticle size on immune cell cytotoxicity and cytokine induction Nanoscale Res Lett 2009 9 12 1409 1420 10.1007/s11671-009-9413-8 20652105 PMC2894345

[B2] Hayashi T Senda M Morohashi H Higashi H Horio M Kashiba Y Nagase L Sasaya D Shimizu T Venugopalan N Tertiary structure-function analysis reveals the pathogenic signaling potentiating mechanism of Helicobacter pylori oncogenic effector CagA Cell Host Microbe 2012 9 20 33 10.1016/j.chom.2012.05.010 22817985

[B3] Hostetter RK Cooper E Coelomocytes as effector cells in earthworm immunity Immunology 1972 9 155 183 10.3109/08820137209022933 4663513

[B4] Engelmann P Palinkas L Cooper EL Nemeth P Monoclonal antibodies identify four distinct annelid leukocyte markers Dev Comp Immunol 2005 9 599 614 10.1016/j.dci.2004.10.008 15784291

[B5] Porchet-Hennere E Dugemont T Fischer A Natural killer cells in a lower invertebrate, Nereis diversicolor Eur J Immunol 1992 9 99 107 1644067

[B6] Cooper RG Kleinschmidt EJ Benchmarking the firm’s critical success factors in new product development J Prod Innovat Manag 1995 9 374 391 10.1016/0737-6782(95)00059-3

[B7] Cooper EL Beschin A, Bilej M, Cooper EL The earthworm: a new model with biomedical applications New Model for Analyzing Antimicrobial Peptides with Biomedical Applications 2002 Amsterdam: IOS 3 26

[B8] Cossarizzra A Ceccarelli D Masine A Functional heterogeneity of an isolated mitochondrial population revealed by cytofluorometric analysis at the single organelle level Exp Cell Res 1996 9 84 94 10.1006/excr.1996.0011 8549677

[B9] Koros WJ Gas separation membranes: needs for combined materials science and processing approaches Micromoles 2002 9 1 13 22

[B10] Valembois P Lassegues M Roch P Formation of brown bodies in the coelomic cavity of earthworm Eisenia fetida andrei and attendant changes in shape and adhesive capacity of constitutive cells Dev Comp Immunol 1992 9 95 101 10.1016/0145-305X(92)90010-A 1499843

[B11] Muravev RA Roogovin VV Fitzpatrick LC Goven AJ Antixenosomes Izv Akad Nauk Ser Biolcheskaia/Rossiiskaia Akademia Nauk 1994 9 197 204

[B12] Adamowicz A Morphology and structure of the earthworm Dendrobena veneta (Lumbricidae) coelomocytes Tissue Cell Cult 2005 9 125 133 10.1016/j.tice.2004.11.002 15748739

[B13] Hayashi Y Engelmann P Foldbjerg R Szabo M Somogyi I Pollak E Earthworms and humans in vitro: characterizing evolutionarily conserved stress and immune responses to silver nanoparticles Environ Sci Technol 2012 9 4166 4173 10.1021/es3000905 22432789

[B14] Scott-Fordsmand JJ Krogh PH Schaefer M Johansen A The toxicity testing of double-walled nanotubes-contaminated food to Eisenia veneta earthworms Ecotoxicol Environ Saf 2008 9 616 619 10.1016/j.ecoenv.2008.04.011 18514310

[B15] Li D Alvarez PJ Avoidance, weight loss, and cocoon production assessment for Eisenia fetida exposed to C_60_ in soil Environ Toxicol Chem 2011 9 2542 2545 10.1002/etc.644 21842489

[B16] Vander Ploeg MJ Baveco JM Vander Hout A Bakker R Rictjens IM Vanden Brink NW Effect of C_60_ nanoparticles exposure on earthworms (Lumbricus rubellus) and implications for population dynamics Environ Pollut 2011 9 198 203 10.1016/j.envpol.2010.09.003 20932615

[B17] Peterson EJ Huang Q Weber JWJ Bioaccumulation of radio-labeled carbon nanotubes by Eisenia foetida Environ Sci Technol 2008 9 3090 3095 10.1021/es071366f 18497171

[B18] Cholewa J Feeney GP Reilly M Sturzenbaum SR Morgan AJ Plytycz B Autofluorescence in eleocytes of some earthworm species Histochem Cytochem 2006 9 65 71 16584095

[B19] Vander Ploeg MJ Vanden Berg JH Bhattacharjee S Dehaan LH Ershov DS Fokkink RG In vitro nanoparticle toxicity to rat alveolar cells and coelomocytes from the earthworm Lumbricus rubellus Nanotoxicology 2012 9 28 37 doi:10.3109/17435390.744857 23102209 10.3109/17435390.2012.744857

[B20] Nel A Xia T Madler L Li N Toxic potential of materials at the nanolevel Science 2006 9 622 627 10.1126/science.1114397 16456071

[B21] Wardak A Gorman ME Swami N Deshpande S Identification of risks in the life cycle of nanotechnology-based products J Indian Ecol 2008 9 3 435 448 10.1111/j.1530-9290.2008.00029.x

[B22] Zha CS Mao HK Hemley RJ Elasticity of MgO and a primary pressure scale to 55 GPa Proc Natl Acad Sci 2000 9 13494 13499 10.1073/pnas.240466697 11095719 PMC17603

[B23] Benn TM Westerhoff P Nanoparticle silver released into water from commercially available sock fabrics Environ Sci Technol 2008 9 11 4133 4139 10.1021/es7032718 18589977

[B24] Kiser MA Westorhoff P Benn T Wang Y Perriz Rivera J Hristovski K Titanium nanomaterial removal and release from wastewater treatment plants Environ Sci Technol 2009 9 6757 6783 10.1021/es901102n 19764246

[B25] Mueller NC Nowak B Environmental impacts of nanosilver Environ Sci Technol 2008 9 4447 4453 10.1021/es7029637 18605569

[B26] Gottschalk F Sonderer T Scholz RW Nowwack B Modelled environmental concentrations of engineered nanomaterials (TiO_2_, ZnO, Ag, CNT, fullerenes) for different regions Environ Sci Technol 2009 9 24 9216 9222 10.1021/es9015553 20000512

[B27] Brousseau KR Dunier M De Guise S Fournier M Lagadic L, Caquet T, Amiard JC, Ramade F Marqueurs immunologiques Biomarqueurs en Ecotoxicologie & Aspects Fondamentaux 1997 Paris: Masson 287 315

[B28] Eyambe SG Goven AJ Fitzpatrick LC Venables BJ Cooper EL A non-invasive technique for sequential collection of earthworm (Lumbricus terrestris) leukocytes during subchronic immunotoxicity studies Lab Anim 1991 9 61 70 10.1258/002367791780808095 2010977

[B29] Brousseau P Fugere N Bernier J Coderre D Nadeau D Poirier G Fournier M Evaluation of earthworm exposure to contaminated soil by cytometric assay of ceolomocytes phagocytosis in Lumbricus terrestris (Oligochaeta) Soil Biol Biochem 1997 9 681 684 10.1016/S0038-0717(96)00029-6

[B30] Brousseau P Payette Y Tryphonas H Blakley B Boermans H Flipo D Fournier M Manual of Immunological Methods 1999 Boca Raton: CRC

[B31] Singh NP Mc Coy MT Tice RR Schneider EL A simple technique for quantitation of low levels of DNA damage in individual cells Exp Cell Res 1988 9 184 191 10.1016/0014-4827(88)90265-0 3345800

[B32] Collins N McManus R Wooster R Mangion J Seal S Lakhani SR Consistent loss of the wild type allele in breast cancers from a family linked to the BRCA2 gene on chromosome 13q12-13 Oncogene 1995 9 1673 1675 7731724

[B33] Yang X Gondikas AP Marinakos SM Auffan M Liu J Hsu-Kim H Mechanism of silver nanoparticle toxicity is dependent on dissolved silver and surface coating in Caenorhabditis elegans Environ Sci Technol 2011 9 1119 1127 22148238 10.1021/es202417t

[B34] Sinha R Li CS Imaging stress- and cue-induced drug and alcohol craving: association with relapse and clinical implications Drug Alcohol Rev 2007 9 25 31 17364833 10.1080/09595230601036960

[B35] Satchell JE Satchell JE Earthworm microbiology Earthworm Ecology: From Darwin to Vermiculture 1983 London: Chapman and Hall 351 365

[B36] Gao H Yang Z Zhang S Cao S Shen S Pang Z Jiang X Ligand modified nanoparticles increases cell uptake, alters endocytosis and elevates glioma distribution and internalization Sci Rep 2013 9 2534 2553 doi:10.1038/srep02534 23982586 10.1038/srep02534 PMC3755284

[B37] Ireland MP Richards KS The occurrence and localisation of heavy metals and glycogen in the earthworms Lumbricus rubellus and Dendrobaena rubida from a heavy metal site Histochenistry 1977 9 153 166 10.1007/BF00567221 845058

[B38] Bystrzejewska-Piotrowska G Asztemborska M Giska I Mikoszewski A Influence of earthworms on extractability of metals from soils contaminated with Al_2_O_3_, TiO_2_, Zn, and ZnO nanoparticles and microparticles of Al_2_O_3_ Pollut Environ Stud 2012 9 2 313 319

[B39] Lunov O Zablostskii V Syrovets T Modeling receptor-mediated endocytosis of polymer-functionalized iron oxide nanoparticles by human macrophages Biomaterials 2011 9 547 555 10.1016/j.biomaterials.2010.08.111 20880574

[B40] Ballarian L Burighel P RGD-containing molecules induce macropinocytosis in ascidian hyaline amoebocytes J Invertebr Pathol 2006 9 124 130 10.1016/j.jip.2005.11.002 16406401

[B41] Franc NC Dimarcq JL Lagueux M Hoffmann J Ezekowitz RA Croquemort, a novel Drosophila hemocyte/macrophage receptor that recognizes apoptotic cells Immunity 1996 9 431 443 10.1016/S1074-7613(00)80410-0 8630729

[B42] Lin CY Zheng QA Huang SJ Kuo NJ Variability of sea surface temperature and warm pool area in the South China Sea and its relationship to the western Pacific warm pool J Oceanogr 2011 9 6 719 724 doi:10.1007/s 10872-011-0072-x 10.1007/s10872-011-0072-x

[B43] Molnar L Engelmann P Somogyi I Mascik LL Pollak E Cold-stress induced formation of calcium and phosphorous rich chloragocyte granules (chloragosomes) in the earthworm Eisenia fetida Comp Biochem Physiol 2012 9 109 209 10.1016/j.cbpa.2012.06.005 22710253

[B44] Beer C Odbjerg R Hayashi Y Sutherland DS Autrup H Toxicity of silver nanoparticle Toxicol Lett 2012 9 3 286 292 10.1016/j.toxlet.2011.11.002 22101214

[B45] Homa J Zorska A Wesolowski D Chadzinska M Dermal exposure to immunostimulants induces changes in activity and proliferation of coelomocytes of Eisenia andrei J Comp Physiol 2013 9 313 322 10.1007/s00360-012-0710-7 PMC3607720 23014884

[B46] Opper B Nemeth P Engelmann P Calcium is required for coelomocyte activation in earthworms Mol Immunol 2010 9 2047 2056 10.1016/j.molimm.2010.04.008 20439116

